# The prognostic significance of circulating tumor cells in head and neck and non‐small‐cell lung cancer

**DOI:** 10.1002/cam4.1832

**Published:** 2018-11-22

**Authors:** Arutha Kulasinghe, Joanna Kapeleris, Rebecca Kimberley, Stephen R. Mattarollo, Erik W. Thompson, Jean‐Paul Thiery, Liz Kenny, Ken O’Byrne, Chamindie Punyadeera

**Affiliations:** ^1^ The School of Biomedical Sciences, Institute of Health and Biomedical Innovation Queensland University of Technology Kelvin Grove Queensland Australia; ^2^ Translational Research Institute Brisbane Queensland Australia; ^3^ Cancer Care Services Princess Alexandra Hospital Woolloongabba Queensland Australia; ^4^ The University of Queensland Diamantina Institute Woolloongabba Queensland Australia; ^5^ Comprehensive Cancer Center Gustave Roussy Villejuif France; ^6^ School of Medicine, Royal Brisbane and Women’s Hospital, Central Integrated Regional Cancer Services, Queensland Health University of Queensland Queensland Australia; ^7^ Princess Alexandra Hospital Brisbane Queensland Australia

**Keywords:** ALK, circulating tumor cells, EGFR, head and neck cancers, liquid biopsy, non‐small‐cell lung cancer

## Abstract

Tumor biopsy is the gold standard for the assessment of clinical biomarkers for treatment. However, tumors change dynamically in response to therapy, and there remains a need for a more representative biomarker that can be assayed over the course of treatment. Circulating tumor cells (CTCs) may provide clinically important and comprehensive tumoral information that is predictive of treatment response and outcome. Blood samples were processed for CTCs from 56 patients using the ClearCell FX system. Captured cells were phenotyped for CTC clusters and markers for immunotherapy (PD‐L1) CTC chromosomal architecture (ALK, EGFR). CTCs were isolated in 11/23 (47.8%) of head and neck cancer (HNC) patients and 17/33 (51.5%) of non‐small‐cell lung cancer (NSCLC) patients. CTCs were determined to be PD‐L1‐positive in 6/11 (54.4%) HNC and 11/17 (64.7%) NSCLC cases, respectively. 3D chromosomal DNA FISH for ALK and EGFR molecular targets showed better resolution than in 2D when imaging CTCs. HNC CTC‐positive patients had shorter progression‐free survival (PFS) (hazard ratio[HR]: 4.946; 95% confidence internal[CI]:1.571‐15.57; *P* = 0.0063), and PD‐L1‐positive CTCs were found to be significantly associated with worse outcome ([HR]:5.159; 95% [CI]:1.011‐26.33; *P* = 0.0485). In the advanced stage NSCLC patient cohort, PFS was not found to be associated with CTCs prior to therapy ([HR]:2.246; 95% [CI]:0.9565‐5.273; *P* = 0.0632), nor the presence of PD‐L1 expression ([HR]:1.646; 95% [CI]:0.5128‐5.283; *P* = 0.4023). This study demonstrated that CTCs are predictive of poorer outcomes in HNC and provides distinct and separate utility for CTCs in HNC and NSCLC, which may be more representative of the disease burden and overall survival than the parameters used to measure them.

## 1 INTRODUCTION

The emerging success of targeted therapy, particularly immune checkpoint blockage, has led to durable responses and prolonged survival in a number of tumor types, including non‐small‐cell lung cancer (NSCLC)^1^, ^2^ and head and neck cancers (HNC).^3^ Given the invasive nature of a tumor biopsy and the limitations of the static snapshot it provides, there remains a critical need for predictive biomarkers to guide patient selection for targeted therapies.^4^ Liquid biopsies may provide an alternative to tissue biopsy, allowing for noninvasive, serial monitoring in real time to assay dynamic tumor changes following selective pressure of targeted treatment.^5^, ^6^

Circulating tumor cells (CTCs) were first described by an Australian Physician, Thomas Ashworth, who observed cells identical to those of the primary cancer in the blood of a patient.^7^, ^8^ Since this discovery, the field remained in its infancy until the last 15 years, largely due to the significant technical challenges associated with capturing these rare cancer cells in a background of billions of normal blood cells. The “needle in a haystack” paradigm was overcome with the improvements in isolation platforms, which have driven the field exponentially.^9^ With the advent of the FDA‐approved CellSearch system (Menarini Silicon Biosystems, Huntingdon Valley, PA), important clinical correlations repeatedly arose between CTC enumeration and survival parameters (overall survival, progression‐free survival) in several tumor types, such as breast, prostate, and colorectal cancers.^10^, ^11^

Despite these profoundly positive prognostic indications, recent studies have demonstrated the inherent bias of EpCAM preselection by the CellSearch system and the potential improvements achieved by epitope dependent platforms.^14^, ^15^ In particular, due to the downregulation of EpCAM‐associated epithelial‐mesenchymal transition (EMT),^17^, ^18^ CTC detection methodologies reliant on EpCAM pre‐enrichment have shown their limitations in capturing only a subset of CTCs.^15^, ^19^, ^20^ Furthermore, a number of studies have demonstrated the presence of CTC clusters,^9^, ^14^, ^21^, ^22^ which may carry a higher potential to metastasize and may have inherent immune evading strategies with the association of immune cells within clusters.^23^ Accordingly, a plethora of alternative CTC enrichment technologies have emerged (eg, immunoaffinity, microfluidics, density gradient centrifugation, microfiltration, acoustophoresis), each with its own advantages and disadvantages.^24^, ^25^ Microfluidic platforms have come to the fore to cater for “label‐free” CTC capture and high‐throughput CTC isolation and analysis.^24^, ^28^, ^29^

We used the ClearCell FX System (Clearbridge Biomedics, Singapore) for CTC isolation in this study, using the CTChip^®^. This system exploits size‐based differences between CTCs and hematopoietic cells by using Dean migration and inertial focusing to achieve CTC separation from cells of the blood. We evaluated CTCs from NSCLC and HNC patients, for a marker used to guide patient selection for immune therapy (PD‐L1), CTC chromosomal architecture using molecular probes (ALK translocations, EGFR), the cell junction component plakoglobin, and the presence of CTC clusters. The study was designed to characterize CTCs and potential subpopulations, from two different cancer types, as potential surrogate markers of tumor aggressiveness and potential targeted/immunotherapy guidance.

## 2 MATERIALS AND METHODS

### 2.1 ClearCell FX system CTC enrichment

The ClearCell FX system utilizes the CTChip^®^, which separates cells based on size (>14 µm) and deformability parameters. The channels in the CTChip^®^ have dimensions that allow CTCs to undergo inertial focusing, while smaller hematologic cells (leukocytes and red blood cells) are affected by the Dean Drag (a_p_/h ~ 0.1 ratio). In the system, diluted, red blood cell depleted blood samples are pumped through the outer inlet, and sheath buffer is pumped through the inner inlet at a higher flow rate to confine the sample stream to the outer wall. As the sample passes through the channel, the total volume of cells initially migrates along the Dean vortex and migrates toward the inner channel. Along the inner walls, the CTCs/CTC clusters (of larger size compared to hematologic cells) focus tightly as they experience inertial lift forces preventing them from migrating under Dean drag. In so doing, the smaller hematologic cells flow along the Dean vortex toward the outer wall. This allows for continuous collection of CTCs at the inner outlet and hematologic cells at the outer outlet.^24^, ^31^

### 2.2 Patient recruitment

This prospective study was conducted across two major academic hospitals in Brisbane, Australia. Ethics approval was obtained from the Metro South Health District Human Research Ethics Committee in accordance with the National Health and Medical Research Council's guidelines (HREC/11/QPAH/331 and HREC/12/QPAH/381) to collect blood samples from the Princess Alexandra Hospital (PAH) and Royal Brisbane and Women's Hospital (RBWH). All methods were performed in accordance with these ethical guidelines and regulations. This study has also been given institutional approval from the Queensland University of Technology Human Ethics Committee (1400000617 and 1100001420). Following written informed consent, 9 mL of blood was collected in K2E vacutainers (EDTA) or Streck tubes from a total of 61 participants. Blood samples were collected from n = 23 HNC patients (Stages I‐IV), n = 33 NSCLC patients (Stage IV), and five normal healthy volunteers (NHV). All HNC and NSCLC patients were treatment naïve at the time of blood collection (Table 1). The treatment regimen for NSCLC is documented in Supplementary Table 1.

**Table 1:** Clinicopathological findings of head and neck (HNC) and non‐small‐cell lung cancer (NSCLC) patient cohorts

Head and neck cancer	N	Non‐small‐cell lung cancer	N
Total	23	Total	33
Gender		Gender	
Male	17 (73.9%)	Male	25 (75.8%)
Female	6 (26.1%)	Female	8 (24.2%)
Age, y		Age, y	
<60	10 (43.5%)	<60	11 (33.3%)
>60	13 (56.5%)	>60	22 (66.6%)
Age range	21‐77	Age range	39‐82
Tumor type		Tumor type	
Oral cavity	9 (39.1%)	NSCLC Adenocarcinoma	30 (90.9%)
Oropharynx	14 (60.9%)	NSCLC SCC	3 (9.1%)
Tumor stage		Tumor stage	
I	4 (17.4%)	IV	33 (100%)
II	3 (13.0%)		
III	4 (17.4%)	Mutation status (tumor)	
IV	12 (52.2%)	EGFR wild type	1 (3.1%)
		EGFR mutation	1 (3.1%)
HPV status		ALK translocation	7 (21.2%)
HPV‐positive	11 (47.8%)	KRAS mutant	1 (3.1%)
HPV‐negative	8 (34.8%)		
Unknown	4 (17.4%)		
CTC findings		CTC findings	
CTC+ (pCK+DAPI+CD45‐)	11/23 (47.8%)	CTC+ (pCK+DAPI+CD45‐)	17/33 (51.5%)
CTC‐ (CD45+DAPI+)	12/23 (52.2%)	CTC‐ (CD45+DAPI+)	16/33 (48.5%)
# Patients with PD‐L1+ CTCs	6/11 (54.5%)	# Patients with PD‐L1+ CTCs	11/17 (64.7%)
# Patients with PD‐L1‐ CTCs	5/11 (45.5%)	# Patients with PD‐L1‐ CTCs	6/17 (35.3%)

CTC‐positive includes single CTCs and CTC clusters. PD‐L1 was evaluated in the CTC‐positive samples and reported as PD‐L1 (positive/negative) if one or more CTCs were PD‐L1‐positive.

### 2.3 The development of a PD‐L1 range

Seven cell lines (4 HNC, 2 NSCLC, and 1 negative control) were used to develop a dynamic range of PD‐L1 expression.^32^ Fadu (ATCC^®^HTB43^TM^) and SCC25 (ATCC^®^CRL1628^TM^) were sourced from the American Type Culture Collection (ATCC™). SCC15 (ATCC^®^CRL1623™) was a generous gift from Dr Glen Boyle (QIMR, Brisbane) and 93‐VU‐147T (CVCL_L895) (HPV‐positive) cell line from Dr Johan de Winter (VU Medical Center, Netherlands). The NSCLC cell lines HCC827 (ATCC^®^CRL2868™) and H460 (ATCC^®^CRL177™) were a generous gift from Prof Derek Richards (QUT, Brisbane). The human chronic myelogenous leukemia K562 (ATCC®CCL243) cells were used as a negative PD‐L1 control (gift from Prof Maher Gandhi, UQDI, Brisbane). Cells were cultured under standard conditions in humidified incubators at 37°C, 5% CO_2_ in RPMI1640‐Glutamax (Life Technologies, Inc) supplemented with 10% fetal bovine serum (FBS) and Penicillin/Streptomycin. Cell line authenticity was confirmed by short tandem repeat (STR) profiling with Stem Elite ™ ID system (Promega) according to manufacturer's instructions. Cell lines were confirmed to be negative for mycoplasma infection by Hoechst staining and PCR. Briefly, the cell lines were transferred onto glass slides, fixed with 4% formaldehyde (Thermo Fisher Scientific) for 10 minutes, permeabilized with 0.2% Triton X‐100 for 5 minutes, and blocked with 10% fetal bovine serum for 1 hour at room temperature. The slides were then stained with anti‐PD‐L1 (1:200 dilution (28‐2) Alexa Fluor^®^647, Abcam]) at 4°C overnight. DAPI was used to visualize nuclear DNA and mounted with Prolong Gold (Molecular Probes, Invitrogen) before coverslipping and imaging. The mean fluorescence intensity was determined per cell line population and analyzed using ImageJ software.

### 2.4 Enrichment of CTCs using the ClearCell FX

7.5 mL of blood was combined with 22.5 mL of red blood cell lysis buffer (Astral Scientific), mixed gently by inverting and left to stand at room temperature for approximately 10 minutes. Cells were spun down at 500 *g* for 10 minutes and the supernatant removed. The pellet was resuspended in 4.3 mL of resuspension buffer (RSB, ClearBridge Biomedics) and loaded onto the ClearCell^®^ FX1 system. The sample was run through the CTC Chip™ FR1, under protocol 1, which is optimal for CTC enumeration and molecular analysis studies. The CTC output was collected and spun down at 300 *g* for 5 minutes prior to cyto‐centrifuging the samples onto glass slides for phenotyping.

### 2.5 CTC immunophenotyping

Circulating tumor cells enriched samples were stained with the CellSearch antibody cocktail (Menarini Silicon Biosystems) targeting pan‐cytokeratin, CD45, and DAPI. Cells were further phenotyped for PD‐L1 (1:200 dilution, Abcam) and gamma‐catenin (1:400 dilution, Cell Signaling) expression. Briefly, the cytospots were incubated with the antibody cocktail of CellSearch Reagents (10 µL staining reagent, 10 µL permeabilization buffer, 10 µL fixation buffer, 10 µL DAPI in 60 µL PBS) at4°C overnight, washed three times in PBS, and air‐dried. The slides were mounted with Prolong Gold mounting medium (Molecular Probes, Invitrogen) to prevent photo‐bleaching and preserve the fluorescent labeled molecules for long‐term storage, cover slipped and imaged on the Zeiss Axio Z2 microscope (Carl Zeiss, Ontario). Results were categorized into CTC‐negative or ‐positive for putative CTCs. Cells were classified as CTCs as previously described.^23^ The mean fluorescence intensity (MFI) of PD‐L1 was determined for each CTC, by subtracting the local background intensity from each CTC measured by mean fluorescence intensity. The expression was compared to known HNC and NSCLC PD‐L1‐positive cell lines and negative control (K‐562).

### 2.6 CTC molecular characterization

Circulating tumor cells slide preparations were fixed in 4% paraformaldehyde and dehydrated via an ethanol series (70%, 85%, and 96%). Slides were treated with RNase (4 mg/mL) (Sigma, USA) and fluorescence in situ hybridization (FISH) carried out using the Vysis LSI ALK break apart probes (Abbott, USA) for NSCLC and EGFR/CEN7 FISH probe mix (DakoCytomation, Denmark) for HNC, and counterstained with DAPI as previously described.^24^, ^33^ The slides were cover slipped and imaged on the Zeiss Axio Imager Z2 microscope.

### 2.7 3D DNA FISH

Scanning of CTCs was performed on the Zeiss Axio Z2 microscope, which captured sequential images on the slides after using fluorescent staining and molecular staining. FISH staining was determined in DAPI^+CD45‐^ cells. FISH parameters (z‐stacking, distance between z‐stacks and exposure times) were optimized for FISH signal identification. FISH scanning parameters were determined to identify a maximum number of signals per enriched CTC by optimization of the z‐stack depths (Range of 5‐45 stacks, distance of 0.1‐0.5 µm between two z‐stacks) and a multi‐exposure protocol for the red and green fluorophores. This is necessary as CTCs tend to have a large nuclear size. The Zen software (Zeiss) was used to interrogate the 2D and 3D images. Deconvolution of the images was performed with the constrained iterative algorithm. Signals were captured by experienced users and the ALK/EGFR status validated by an experienced cytogeneticist.

### 2.8 ALK FISH parameters

In cells with native ALK status, the overlapping 3’ (red) and 5’ (green) signals produced a fused 3’5’ (yellow signal). The characteristic ALK translocation was identified when a split of the 3’ (red) and 5’ (green) signal was observed, or a single 3’ (red) signal was observed with a distance of more than two signal diameters.

### 2.9 EGFR FISH parameters

EGFR status was scored as the ratio of the number of EGFR signals (red) to CEP7 (green) signals. An increase in copy numbers of the EGFR gene is represented by higher numbers of red to green signals. A highly amplified result was defined as EGFR: CEP7 ratio of >3 or EGFR gene clusters and non‐amplified result as an EGFR: CEP7 ratio <2.^33^, ^34^

### 2.10 ALK CDx assay

The VENTANA ALK (D5F3) companion diagnostic test was used to determine ALK protein in formalin‐fixed, paraffin‐embedded (FFPE) tissue from corresponding patient samples and stained with a BenchMark XT automated staining instrument.

### 2.11 Statistical analysis

Patients were categorized by the presence or absence of CTCs and by response to treatment (complete, partial, stable disease, progressive disease). The primary objective of the study was to determine the association between CTCs (prior to therapy) and progression‐free survival (PFS). Kaplan‐Meier method was used to estimate event‐time distributions and compared by the log‐rank test. A *P* < 0.05 was considered statistically significant.

## 3 RESULTS

**Figure 1:**
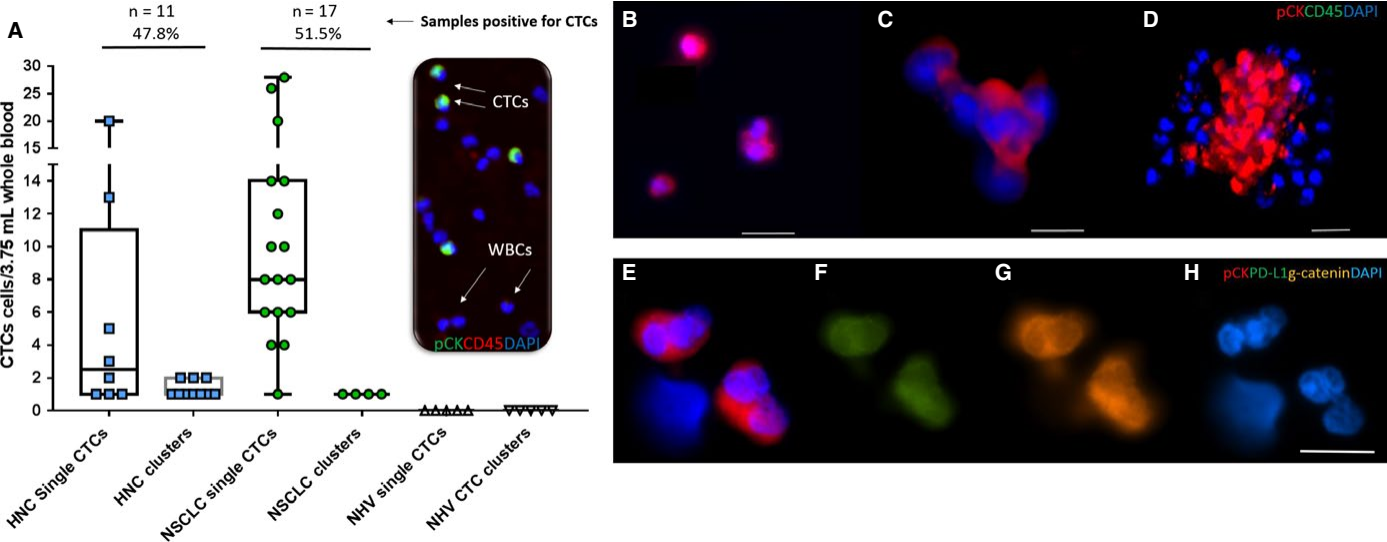
(A) CTC findings in the head and neck cancer (HNC, n = 23) and non‐small‐cell lung cancer (NSCLC, n = 33)—CTC distributions per 3.75 mL blood samples. Single CTCs and clusters of CTCs (pan‐cytokeratin+CD45‐DAPI+cells) were distinguishable from the white blood cell (WBC) (CD45+DAPI+). CTCs were identified in 11/23 HNC samples (47.8%, range 1‐20 single CTCs/3.75 mL blood, 1‐2 CTC clusters) and 17/32 NSCLC samples (53.1%, range 1‐28 CTCs/3.75 mL blood, 1 CTC cluster). CTC‐like events were not observed in the normal healthy volunteer (NHV) samples (n = 5). [B‐D] Examples of CTCs types detected (B) single, doublets (C) CTC cluster with 4‐5 cells (D) Multicellular cluster of CTCs. [E‐H] CTC clusters characterized for (E) pan‐cytokeratin (red) and nucleus stain DAPI (blue) (F) PD‐L1 (green) (G) gamma‐catenin (plakoglobin) (orange) (E) DAPI imaged on the Zeiss Axio Imager Z2 microscope. Scale bar represents 50 µm

Peripheral blood samples were collected from n = 23 head and neck cancer patients (early‐advanced stage of disease; Stages I‐IV) and n = 33 NSCLC patients (Stage IV). CTCs and CTC clusters were successfully isolated using the ClearCell FX (Figure S1). CTCs (either single cells/CTC clusters) were isolated in 11/23 (47.8%) HNC patients (range 1‐20 single CTCs/3.75 mL and 1‐2 CTC clusters). CTCs were positive in 1/4 Stage I, 2/3 Stage II, 1/4 Stage III, 7/12 Stage IV HNC. In NSCLC, CTCs were isolated in 17/33 (51.5%) of patients (range 1‐28 CTCs/3.75 mL, 1 CTC cluster; all Stage IV; Figure 1). No CTC‐like events were found in the five normal healthy volunteer samples run on the ClearCell FX. The clinicopathological findings are presented in Tables 1 and S1.

### 3.1 Eligibility for immunotherapy

While tumor tissue was not evaluable for PD‐1/PD‐L1 for this study, 12 NSCLC patients were selected for immunotherapy based on disease progression after conventional first‐line therapy. None of the HNC patients were given immunotherapy.

### 3.2 CTC findings

**Figure 2:**
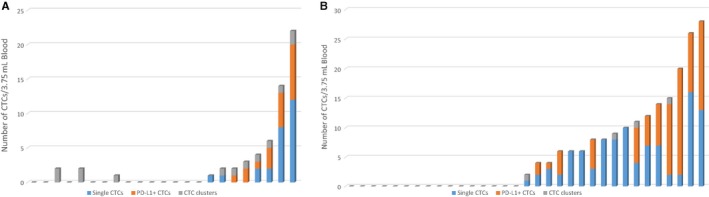
The distribution of CTCs per 3.75 mL of blood is shown in (A) head and neck cancer (n = 23) and (B) non‐small‐cell lung cancer cohorts (n = 33). The single CTCs per patient are shown in blue, CTC‐positive for PD‐L1 in orange and CTC clusters in gray

**Figure 3:**
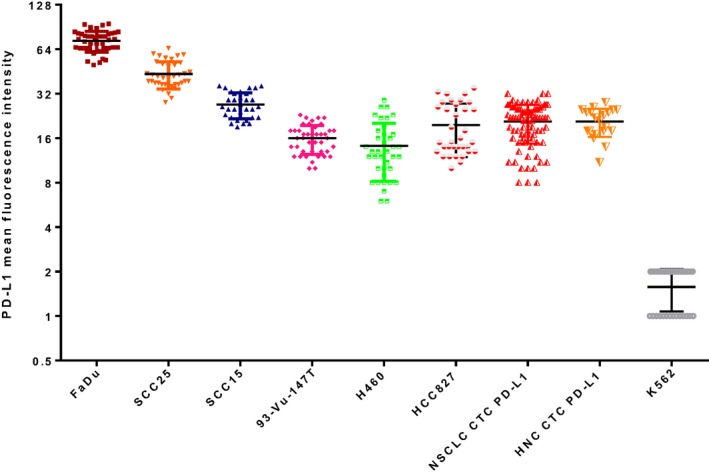
PD‐L1 status measured by mean fluorescence intensity (MFI) on a log2 scale for head and neck cancer (HNC) cell lines (Fadu, SCC25, SCC15, 93‐VU‐147T), non‐small‐cell lung cancer (NSCLC) cell lines (H460, HCC827), negative control (K652), and NSCLC and HNC patient CTCs

PD‐L1 was found to be expressed (minimum of 1 CTC staining positive by immunofluorescence) in 6/11 (54.4%) HNC samples. Only in two HNC cases were all the CTCs PD‐L1‐positive (Figure 2A). PD‐L1 was found to be positive in 11/17 (64.7%) NSCLC CTC‐positive samples (Figure 2B). There were no NSCLC samples where all CTCs were PD‐L1‐positive. Of the 12 NSCLC patients selected for immunotherapy (nivolumab), 9/12 (75%) had detectable CTCs of which 6/9 (66.7%) had PD‐L1‐positive CTCs. The mean fluorescence intensity (MFI) of PD‐L1 expression on HNC and NSCLC CTCs in comparison with known HNC/NSCLC cell lines is shown in Figure 3. CTC clusters were identified in 10/23 HNC samples and 4/33 NSCLC samples (Figure 2). CTC clusters were found in all NSCLC cases where single CTCs were present; however, in HNC, three patients presented with only CTC clusters and seven patient samples presented with both single and clustered CTCs. Gamma‐catenin (plakoglobin) was assessed in a subset (n = 4) HNC and (n = 2) NSCLC cluster samples, and showed plasma membrane localization in the subset of samples.

### 3.3 Molecular analysis

**Figure 4:**
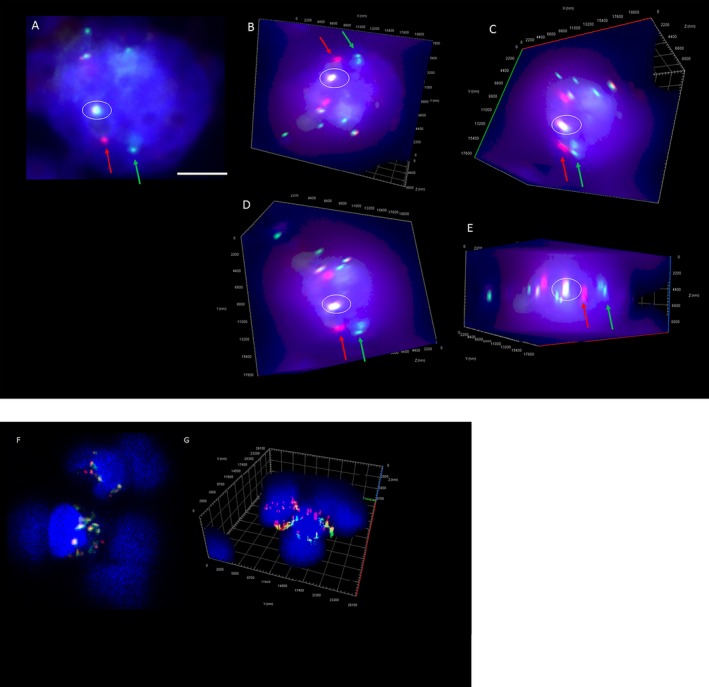
(A) 2‐dimensional image of an ALK‐rearranged NSCLC CTC. (Series B‐E) 3D volume Z‐stack images of CTC (A) rotated about its axis showing the depth of the cell and the additional signals found when imaging by z‐stacking (minimum 45 slices). The circled area depicts the fusion (yellow) signal and the location of the split signal of the 3’ (red) and 5’ (green). Scale bar represents 5 µm. (F) 2‐dimensional image of an EGFR amplified HNC CTC. (G) 3D volume of Z‐stack images of CTC showing the additional signals found when imaging by z‐stacking. The ratio of EGFR (red) to CEP‐7 (green) shows EGFR amplification in these cells

Six NSCLC patients had ALK‐positive tumors, confirmed by ALK protein staining (ALK CDx Assay) and ALK‐DNA FISH. Four of the six NSCLC patients had detectable CTCs. ALK was assessed by DNA FISH in the CTCs and found to be translocated in all four CTC‐positive samples (minimum of one ALK‐rearranged CTC/3.75 mL blood). ALK signals were assessed in 2‐dimensional and 3‐dimensional stacks to determine additional signals, which were spatially orientated through the nuclear volume (Figure 4). The 3D resolution of the nuclear volume showed higher resolution of the ALK split signal compared to imaging in 2D. Rotation of the cell about its axis (Figure 4B‐E) allowed for confirmation of the split signal. EGFR gene amplification was confirmed in a subset of HNC CTC‐positive samples (n = 6, minimum of one amplified CTC/3.75 mL blood). EGFR signal to CEP‐7 signal was spatially assessed within the nuclear area (Figure 4F‐G). Upon 3D imaging of the CTC nuclei, the individual signals of the EGFR and CEP‐7 had a higher resolution and separation of individual signals (Figure 4).

### 3.4 Association of CTC with progression‐free survival in HNC and NSCLC

**Figure 5:**
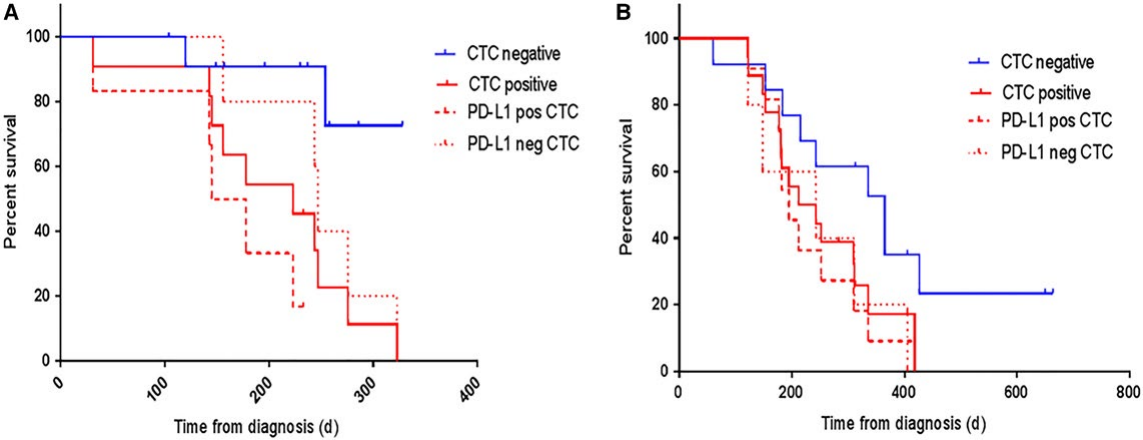
Kaplan‐Meier curves for progression‐free survival (PFS) according to the presence/absence of CTCs for (A) head and neck cancer patients with detectable CTCs (hazard ratio [HR]: 4.946; 95% confidence internal [CI]: 1.571‐15.57; *P* = 0.0063) and PD‐L1‐positive CTCs ([HR]: 5.159; 95% [CI]: 1.011‐26.33; *P* = 0.0485) (B) non‐small‐cell lung cancer patients who were CTC‐positive ([HR]: 2.246; 95% [CI]: 0.9565‐5.273; *P* = 0.0632) and PD‐L1‐positive CTCs ([HR]:1.646; 95% [CI]:0.5128‐5.283; *P* = 0.4023). Patients were CTC‐positive either by the presence of single CTCs and/or CTC clusters. Red solid line (CTC‐positive), red dash line (PD‐L1‐positive CTCs), red dotted line (PD‐L1‐negative CTCs), blue solid line (CTC‐negative)

Kaplan‐Meier survival analysis was performed on the HNC and NSCLC patient cohorts with respect to the baseline CTC findings prior to treatment. CTC‐positive cases were determined as either single or clusters of CTCs. As shown in Figure 5A, HNC patients with CTC‐positive counts had shorter PFS than patients with the absence of CTCs (hazard ratio [HR]: 4.946; 95% confidence internal [CI]: 1.571‐15.57; *P* = 0.0063), and the PD‐L1 positivity in the CTCs was found to be significant ([HR]: 5.159; 95% [CI]: 1.011‐26.33; *P* = 0.0485). In NSCLC, PFS was not associated with presence of CTCs ([HR]: 2.246; 95% [CI]: 0.9565‐5.273; *P* = 0.0632), nor CTC PD‐L1 expression status ([HR]:1.646; 95% [CI]:0.5128‐5.283; *P* = 0.4023) (Figure 5B).

## 4 DISCUSSION

While there are promising data from the CheckMate 141 trial for HNC, and from CheckMate 017 and CheckMate 057 for NSCLC showing survival benefits in nivolumab‐treated patients compared to standard therapy, there remains an urgent need for biomarkers to stratify patient responders from non‐responders.^3^, ^35^ This has been compounded by the poor predictive value of PD‐L1 expression levels in tumor tissue as shown in the Blueprint PD‐L1 IHC Assay comparison project.^36^, ^37^ Moreover, at ASCO 2018, it was made clear from the Keynote 042 study that the broad groupings by PD‐L1 expression in tumor tissue do not allow researchers to predict benefit from pembrolizumab for patients with specific PD‐L1 expression. This has been attributed to the heterogeneity found within tumors, antibody affinities, limited specificity, or variations in target epitopes.^37^ Moreover, the tumor changes dynamically during the course of treatment and a static snapshot by means of a tumor biopsy for the purposes of PD‐L1 assessment may not represent a continuously adapting tumor landscape in response to targeted therapy. Liquid biopsy may provide a real‐time assessment of an ever‐changing tumor.^38^, ^39^

Circulating tumor cells may provide an alternative to tissue biopsy, by representing cells from both the primary and secondary sites which may be more representative of a dynamically changing tumor at a given time. The purpose of this study was to evaluate and characterize CTCs from tumor types where PD‐L1 status may be clinically important, as in HNC and NSCLC. The Clearbridge FX platform demonstrated that size‐ and deformability‐based sorting of CTCs captured baseline CTC populations of clinical significance as shown by the survival data. CTC clusters were also found in both tumor types and expressed plakoglobin, an important cell junction component, which has been shown to be involved in CTC cluster formation and the formation of distant metastasis more readily than single CTCs.^22^, ^23^, ^41^ For the HNC cohort, there was a significant difference in PFS between patients who were CTC‐positive compared to the absence of CTCs. While PFS showed no significant difference (*P* = 0.0632) in the NSCLC cohort, this may be achieved with a larger sample size. Another cofounding factor may be the advanced stage of disease in the NSCLC patients (all Stage IV), where the prognosis is generally poor. Additional factors may, in part, be due to a lower tumor mutation burden and/or the sequence of treatment (chemo/immune therapy).^42^, ^43^

The study provides preliminary data to support that PD‐L1 is evaluable on HNC and NSCLC CTCs, consistent with a number of recent studies.^5^, ^6^, ^29^, ^45^ CTCs have been shown to have a higher PD‐L1 positivity compared to tumor tissue.^46^ In this study, the patient CTC PD‐L1 expression levels were found to be comparable to known cell lines. Moreover, PD‐L1‐positive CTCs at baseline associated with a worse PFS in HNC. In the study by Strati and colleagues in HNC, patients with CTCs overexpressing PD‐L1 at the end of treatment had shorter PFS and OS.^6^ Similar findings were observed by Guibert et al^46^ in NSCLC, where PD‐L1+ CTCs were seen in all patients at progression. Moreover, the presence of high PD‐L1+ CTCs associated with a poorer patient outcome.^47^ While outside the scope of this study, longitudinal blood sampling after treatment could give important insights into the role/persistence of PD‐L1‐positive CTCs.^29^ In the longitudinal follow‐up study by Nicolazzo et al, the authors demonstrated that all patients with PD‐L1‐negative CTCs obtained a clinical benefit, whereas patients with PD‐L1‐positive CTCs experienced progressive disease. This may be representative of a PD‐L1‐positive CTC population that plays a role in immune evasion and therapy escape.^5^, ^48^

In NSCLC, a threshold of 15% ALK‐rearranged cells is used when determining whether a tumor is ALK‐positive.^49^ However, no such thresholds currently exist for CTCs.^50^ While our data are consistent with a number of studies having reported on the presence of ALK rearrangement in CTCs,^49^, ^50^ the spatial distribution of the ALK signal for CTCs has not been reported. The comparison of CTC chromosomal architecture by 3D DNA FISH (ALK, EGFR) showed an underestimation when imaging in 2D conditions. While this depended on the minimal distance between two spots, as with the ALK split signal, this could be overcome by imaging in the XY and YZ planes. The sole measurement of molecular signals in 2D could lead to erroneous interpretation of CTCs.^53^

A limitation of this study is that a comparative tumor PD‐L1 profile would have been desirable to compare CTC expression with tumor tissue. Nonetheless, given the highly variable and heterogeneous nature of PD‐L1 expression levels in tumors, and the fact that some PD‐L1‐positive tumors do not respond to anti‐PD‐1‐directed immunotherapy, a more representative biomarker is needed to determine selection for immunotherapy. Tumors with a high tumor mutation burden (TMB) are thought to lead to an increase in number of neo‐antigens and in turn elicit a more pronounced immune response, giving a greater likelihood of response to immunotherapy.^54^, ^55^

## 5 CONCLUSION

This study demonstrates the potential utility of CTCs in HNC and NSCLC and their applications in the tumor types. While CTCs and the presence of PD‐L1 are predictive of outcome in HNC, this is not the case for NSCLC, which has been known to have a poorer prognosis compared to HNC. Therefore, there remains a distinct separation in the utility of CTCs in the two tumor types, where CTC tracking over time may be more predictive of treatment outcomes in NSCLC.

## CONFLICT OF INTEREST

No conflict of interest exists.
