# Supplementary figures and images for: The prognostic significance of circulating tumor cells in head and neck and non‐small‐cell lung cancer
Source: Cancer Med. 2018 Nov 22;7(12):5910–9. doi: 10.1002/cam4.1832 (PMC6308060; doi:10.1002/cam4.1832)

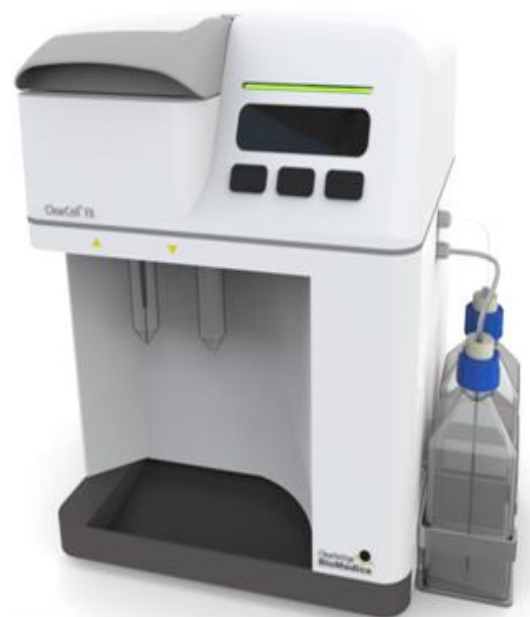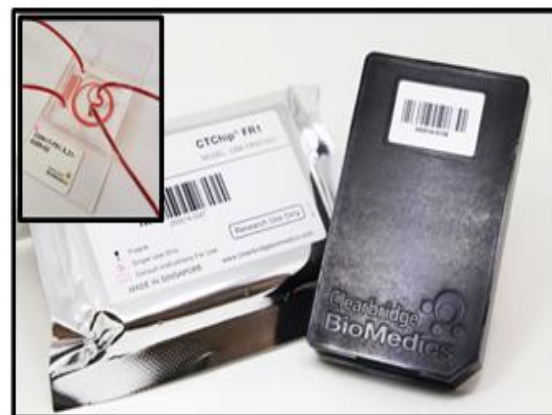

Supplement: Supplementary file 1 [file CAM4-7-5910-s001.pdf]
