# Supplementary material for: The prognostic significance of circulating tumor cells in head and neck and non‐small‐cell lung cancer
Source: Cancer Med. 2018 Nov 22;7(12):5910–9. doi: 10.1002/cam4.1832 (PMC6308060; doi:10.1002/cam4.1832)
Supplement: Supplementary file 2 [file CAM4-7-5910-s002.docx]

| Pt # | Sex | Age Range | NSCLC type | Primary tumour  mutations | 1^st^ line TX | Immunotherapy | CTCs | PD-L1+ |
| --- | --- | --- | --- | --- | --- | --- | --- | --- |
| 1 | M | 45-50 | Adeno | ALK+ | Chemo | Nivolumab | + | + |
| 2 | M | 65-70 | Adeno |  | CRT | Nivolumab | - | - |
| 3 | M | 40-45 | Adeno |  | Chemo |  | - | - |
| 4 | F | 65-70 | Adeno |  | Chemo |  | - | - |
| 5 | M | 65-70 | Squamous |  | CRT | Nivolumab | - | - |
| 6 | F | 50-55 | Adeno | KRAS | CRT |  | + | + |
| 7 | F | 70-75 | Adeno |  | CRT |  | - | - |
| 8 | F | 70-75 | Adeno |  | Surgery |  | - | - |
| 9 | M | 70-75 | Adeno |  |  |  | - | - |
| 10 | M | 80-85 | Adeno |  | CRT |  | - | - |
| 11 | M | 70-75 | Adeno | EGFR WT | Chemo |  | - | - |
| 12 | M | 35-40 | Adeno | ALK+ | Chemo |  | + | + |
| 13 | M | 40-45 | Adeno | ALK+ | Chemo | Nivolumab | + | + |
| 14 | M | 65-70 | Adeno |  | Chemo |  | + | - |
| 15 | F | 55-60 | Adeno |  | Chemo | Nivolumab | + | + |
| 16 | M | 70-75 | Squamous |  | CRT | Nivolumab | + | - |
| 17 | F | 65-70 | Adeno |  | CRT | Nivolumab | + | + |
| 18 | M | 60-65 | Adeno |  | Chemo | Nivolumab | + | + |
| 19 | M | 75-80 | Adeno |  | Chemo | Nivolumab | + | - |
| 20 | M | 70-75 | Adeno | EGFR del19 | TKI |  | - | - |
| 21 | M | 65-70 | Adeno |  | Chemo |  | + | - |
| 22 | M | 60-65 | Squamous |  | CRT | Nivolumab | + | - |
| 23 | M | 75-80 | Adeno |  | Chemo |  | + | - |
| 24 | M | 45-50 | Adeno | ALK+ |  |  | - | - |
| 25 | F | 50-55 | Adeno | ALK+ |  |  | - | - |
| 26 | F | 65-70 | Adeno |  | Chemo |  | + | + |
| 27 | M | 60-65 | Adeno |  | CRT |  | + | + |
| 28 | M | 55-60 | Adeno |  |  |  | - | - |
| 29 | M | 60-65 | Adeno |  |  |  | - | - |
| 30 | M | 50-55 | Adeno | ALK+ |  |  | - | - |
| 31 | M | 55-60 | Adeno |  | Chemo | Nivolumab | + | + |
| 32 | M | 65-70 | Adeno | ALK+ | CRT |  | + | - |
| 33 | M | 60-65 | Adeno |  | Chemo | Nivolumab | - | - |

**Supplementary Table 1.** NSCLC patient cohort, clinicopathological features, mutational status, treatment and CTC parameters. 33 blood samples were analysed from 33 NSCLC patients; 30/33 were adenocarcinoma, 3/33 were squamous cell carcinoma. Adeno: adenocarcinoma, WT: wildtype, TX: treatment, chemo: chemotherapy, TKI: tyrosine kinase inhibitor
